# A cost-effectiveness analysis of intraoperative autologous transfusion in adolescent idiopathic scoliosis surgery: a single-centre retrospective study

**DOI:** 10.1186/s12871-023-02180-3

**Published:** 2023-06-17

**Authors:** Wen Chi, Zhenguo Luo, Zanqing Wu, Jianhong Hao

**Affiliations:** 1 Department of Operating room, HongHui Hospital, Xi’an JiaoTong University, Xi’an, Shaanxi Province China; 2 Department of Anaesthesiology, HongHui Hospital, Xi’an JiaoTong University, Xi’an, Shaanxi Province China

**Keywords:** Autologous transfusion, Idiopathic scoliosis, Cost-effectiveness analysis, Blood loss

## Abstract

**Background:**

Intraoperative autologous transfusion (IAT) has been used in scoliosis surgery for decades; however, its cost-effectiveness remains debatable. This study aimed to evaluate the cost-effectiveness of IAT in adolescent idiopathic scoliosis (AIS) surgery and identify risk factors of massive intraoperative blood during this surgery.

**Methods:**

The medical records of 402 patients who underwent AIS surgery were reviewed. The patients were divided into different groups according to the intraoperative blood loss volume (group A: ≥500 to < 1000 mL, B: ≥1,000 to < 1,500 mL, and C: ≥1,500 mL) and whether IAT was used (i.e., IAT and no-IAT groups). The volume of blood loss, volume of transfused allogeneic red blood cells (RBC), and RBC transfusion costs were analysed. Univariate and multivariate logistic regression analyses were used to identify the independent risk factors of massive intraoperative blood loss (≥ 1,000 mL and ≥ 1,500 mL). A receiver operating characteristic (ROC) curve was used to analyse the cut-off values of the factors contributing to massive intraoperative blood loss.

**Results:**

In group A, no significant difference was observed in the volume of allogeneic RBC transfused during and after procedure between the IAT and no-IAT groups; however, total RBC transfusion costs was significantly higher in the IAT group. In groups B and C, the patients in the IAT group compared with those in the no-IAT group had a lower volume of allogeneic RBC transfused during the operation and on the first day after the operation. However, in group B, the total RBC transfusion cost in the patients who used IAT was significantly higher. In group C, total RBC transfusion cost in the patients who used IAT was significantly lower. The number of fused vertebral levels and Ponte osteotomy were found to be independent risk factors for massive intraoperative blood loss. ROC analysis showed that more than eight and 10 fused vertebral levels predicted ≥ 1,000 mL and ≥ 1,500 mL intraoperative blood loss, respectively.

**Conclusion:**

The cost-effectiveness of IAT in AIS was related to the volume of blood loss, and when the blood loss volume was ≥ 1,500 mL, IAT was cost-effective, drastically reducing the demand for allogeneic RBC and total RBC transfusion cost. The number of fused vertebral levels and Ponte osteotomy were independent risk factors for massive intraoperative blood loss.

## Background

Adolescent idiopathic scoliosis (AIS) is a common spinal deformity, and posterior instrumentation and fusion is a common method for treating AIS [1]. However, the operation is complicated and causes massive intraoperative blood loss (650–2,839 mL) [2–4].

Intraoperative autologous transfusion (IAT) is a technique used for salvaging and reinfusing red blood cells (RBC). IAT has been used in scoliosis surgery for decades; however, its cost-effectiveness remains debatable. Previous studies have discovered that using IAT in scoliosis surgery was cost-effective [5–8]. However, some recent studies revealed that IAT was ineffective in reducing the requirement for allogeneic RBC transfusions [9, 10]. Therefore, determining the cost-effectiveness of IAT warrants further study, especially considering that since the onset of the coronavirus disease pandemic, a shortage in blood supplies has occurred [11].

We conducted a retrospective study to analyse the cost-effectiveness of IAT regarding different volumes of blood loss and to identify risk factors of massive intraoperative blood loss in patients who underwent AIS surgery.

## Methods

### Ethics

The Institutional Review Board of Honghui Hospital, Xi’an Jiao Tong University, approved this retrospective study. Patient information was anonymized; therefore, the need for informed consent was waived by the Institutional Review Board of Honghui Hospital, Xi’an Jiao Tong University.

### Participants

The medical records of 402 patients who underwent AIS surgery at Honghui Hospital between January 10, 2012, and December 31, 2022, were reviewed. Patients with less than 500 mL of blood loss, and those with notable data loss were excluded.

### Surgery and blood transfusion

All patients underwent total intravenous anaesthesia. In the course of the operation, controlled hypotension was performed in all patients to maintain a systolic pressure level of 90 ± 5 mmHg. After disinfection, the skin, subcutaneous tissue, and fascia were cut in turn, and the spinous process, lamina, as well as intervertebral space of the corresponding segment, were exposed, followed by the insertion of the pedicle screws. X-ray fluoroscopy confirmed the position of the pedicle screws, and the malpositioned screws were adjusted. Depending on the location and degree of scoliosis, partial resection of the spinous processes, laminae, and articular processes was selected. Subsequently, the connecting rods were installed and rotated to correct the scoliosis, pressurize, or expand properly as required. X-ray fluoroscopy was performed again to confirm the correct position of the pedicle screw rod. Finally, the nuts were tightened to fix the connecting rod, and facet fusion was performed with autogenous iliac crest bone, which was harvested from the posterior iliac crest at the beginning of the procedure. The drainage tube was placed, after which, each layer of tissue and skin was sutured successively.

None of the patients received erythropoietin and iron therapy during the perioperative period. In all cases, transfusion was administered when blood loss was > 20% of the total blood volume, haemoglobin level was < 7.0 g/dL, or persistent hypotension or tachycardia rate was > 20% from baseline after adequate fluid resuscitation [12–15]. IAT was performed using the Haemonetics Cell Saver® 5+ (Haemonetics, Braintree, MA, USA). All blood loss from the time of skin incision until wound closure was collected by a negative-pressure system (-100mmHg). Heparin (25 U/mL) was added to 0.9% normal saline to prevent coagulation. Autologous blood was washed in 225 mL centrifuge bottles at a centrifuge speed of 5,650 revolutions per minute (rpm) with different washing pump speeds of 300, 500, or 1,000 mL/min (according to the rate of bleeding), which required 1,000 mL of solution. Subsequently, the washed autologous RBCs were transported to a sterile reinfusion bag and transfused to the patient intraoperatively.

### Charges

The cost per unit of allogeneic RBC transfusion was 63.42 USD, including ABO and Rh blood typing, antibody screening, cross-matching, RBC packaging, white blood cell filtration, and storage expense. The cost of IAT per surgery was 243.83 USD, including labour and material costs (Table 1).

**Table 1:** The cost of allogeneic RBC and IAT

	Cost USD
**Allogeneic red blood cell (RBC)**	
ABO and Rh blood typing	2.78
Antibody screening	5.40
Cross matching	4.63
RBC packaging	32.40
White blood cell filtration	16.98
Storage expense	1.23
Total	63.42
**Intraoperative autologous transfusion (IAT)**	
Labour	12.34
Material	231.49
Total	243.83

### Data collection

All data were obtained from the medical records, and the indicators included sex, age, height, weight, Cobb angle of main bending, perioperative haemoglobin and haematocrit level, surgical duration, number of fused vertebral levels, osteotomy pattern, total infused fluid, total infused plasma, urine volume, volume of intraoperative blood loss, volume of drainage after surgery, volume of autologous blood transfusion, volume of allogeneic RBC transfused during and after procedure, allogeneic RBC transfusion-related costs (including allogeneic RBC transfusion-related costs during and after procedure), and total RBC transfusion cost (including allogeneic RBC transfusion-related and IAT-related costs).

Depending on the volume of blood loss during the operation, the patients were divided into three groups: A (≥ 500 to < 1000 mL), B (≥ 1,000 to < 1,500 mL), and C (≥ 1,500 mL). Depending on whether IAT was used, each group was further divided into IAT and no-IAT groups.

### Statistical analysis

All data were analysed using SPSS® for Windows (version 18, SPSS, Inc., Chicago, IL, USA). Variables with normal distribution were expressed as means ± standard deviations (SD) and compared using two independent sample t-tests. Counting data were reported as numbers and compared using the χ2 test. Univariate and multivariate logistic regression analyses were used to identify the independent risk factors of massive intraoperative blood loss (≥ 1,000 mL and ≥ 1,500 mL), and factors with a P < 0.05 in the univariate analysis were selected for logistic multivariate regression analysis. A receiver operating characteristic (ROC) curve was used to analyse the cut-off values of the factors contributing to massive intraoperative blood loss. Statistical significance was set at P < 0.05.

## Results

### Basic demographic characteristics of all participants

This study included 402 patients, including 264 (65.67%) women and 138 (34.33%) men, with an average age of 12.87 years (SD, 2.06; range, 9–19), average height of 151.58 cm (SD, 12.43; range, 121.00–179.00), and average weight of 41.03 kg (SD, 9.66; range, 18–63).

In groups A, B, and C, no significant differences were observed in sex, age, height, weight, Cobb angle, and preoperative haemoglobin and haematocrit level between patients who received IAT and those who did not (Table 2).

**Table 2:** Basic demographic characteristics of all participants

	Group A			Group B			Group C		
	IAT group	No-IAT group	*P*	IAT group	No-IAT group	*P*	IAT group	No-IAT group	*P*
N	59	89		88	74		52	40	
Sex			0.250			0.907			0.373
Male	20	29		29	21		21	18	
Female	39	60		59	53		31	22	
Age, year	13.66 ± 2.85	12.66 ± 2.38	0.104	12.00 ± 1.58	12.45 ± 1.06	0.145	13.20 ± 2.30	13.74 ± 1.11	0.343
Height, cm	144.33 ± 14.95	148.77 ± 11.97	0.158	151.22 ± 11.65	152.14 ± 8.88	0.699	157.40 ± 15.31	157.75 ± 5.04	0.922
Weight, kg	37.13 ± 7.70	39.28 ± 7.28	0.223	40.38 ± 11.45	43.14 ± 9.16	0.248	45.40 ± 10.51	45.00 ± 3.62	0.871
Cobb angle, degree	55.58 ± 15.86	56.01 ± 21.98	0.873	61.04 ± 20.57	60.14 ± 18.65	0.735	67.00 ± 17.45	65.43 ± 20.54	0.856
Preoperative hemoglobin, g/dL	13.53 ± 1.21	13.32 ± 1.62	0.544	13.35 ± 1.01	13.07 ± 0.51	0.134	13.58 ± 1.02	14.12 ± 0.76	0.054
Preoperative hematocrit, %	39.26 ± 3.54	39.13 ± 2.04	0.731	40.01 ± 0.45	39.62 ± 4.501	0.550	39.72 ± 1.94	39.786 ± 3.33	0.331

### Intraoperative and postoperative conditions of all participants

In groups A, B, and C, no significant differences were observed in the number of fused vertebral levels, osteotomy pattern, surgical duration, total fluid infusion, total plasma infusion, urine volume, volume of intraoperative blood loss, volume of drainage after surgery, and postoperative haemoglobin and haematocrit values between patients who received IAT and those who did not (Table 3).

**Table 3:** Intraoperative and postoperative conditions of all participants

	Group A			Group B			Group C		
	IAT group	No-IAT group	*P*	IAT group	No-IAT group	*P*	IAT group	No-IAT group	*P*
N	59	89		88	74		52	40	
Number of vertebral levels fused	6.99 ± 2.11	7.38 ± 1.99	0.691	8.99 ± 2.11	8.28 ± 1.99	0.701	12.01 ± 2.54	12.56 ± 2.01	0.595
Osteotomy pattern			0.121			0.073			0.091
Fusion without osteotomy, n	56	84		70	59		33	27	
Smith-Peterson osteotomy, n	3	5		13	10		9	7	
Ponte osteotomy, n	0	0		5	5		9	6	
Duration of the surgery, h	6.10 ± 1.03	6.34 ± 1.65	0.495	6.54 ± 2.34	6.16 ± 1.76	0.426	8.10 ± 1.96	8.50 ± 2.44	0.546
Total fluid infusion, ml	2375.00 ± 478.63	2456.11 ± 593.59	0.433	2935.71 ± 789.03	3036.11 ± 542.13	0.547	3900.00 ± 762.56	3806.25 ± 1267.55	0.411
Total plasma infusion, ml	166.66 ± 182.51	155.55 ± 127.13	0.756	244.44 ± 158.91	257.14 ± 209.03	0.758	320.00 ± 163.29	350.00 ± 223.60	0.605
Urine volume, ml	833.33 ± 258.75	922.22 ± 431.11	0.314	900.00 ± 293.87	885.71 ± 299.15	0.831	1180.00 ± 394.75	1325.00 ± 443.52	0.252
Volume of intraoperative blood loss, ml	700.00 ± 83.04	711.11 ± 148.43	0.710	1155.55 ± 117.85	1171.42 ± 89.34	0.651	1780.00 ± 208.16	1750.00 ± 170.13	0.605
Volume of autologous blood transfused, ml	240.00 ± 41.52	/		405.55 ± 107.77	/		640.00 ± 122.47	/	
Volume of allogeneic RBC transfused, unit									
During operation	2.66 ± 0.95	3.00 ± 1.16	0.198	3.55 ± 1.27	4.28 ± 0.71	0.003*	5.00 ± 2.04	8.50 ± 1.70	< 0.001*
Day 1 postoperation	1.07 ± 0.35	1.11 ± 0.43	0.089	1.37 ± 0.68	2.01 ± 0.76	0.022*	2.14 ± 1.05	2.98 ± 0.99	0.017*
Day 2 postoperation	0.77 ± 0.21	0.82 ± 0.11	0.372	1.00 ± 0.46	1.09 ± 0.67	0.476	1.27 ± 0.76	1.30 ± 0.86	0.099
Volume of drainage, ml									
Day 1 postoperation	199.44 ± 17.56	202.49 ± 21.12	0.781	358.75 ± 24.21	367.78 ± 19.68	0.088	555.76 ± 38.87	579.01 ± 54.42	0.081
Day 2 postoperation	69.67 ± 11.24	73.78 ± 15.56	0.356	145.76 ± 14.86	157.81 ± 15.62	0.101	203.65 ± 19.77	211.57 ± 14.99	0.077
Hemoglobin, g/dL									
Day 1 postoperation	11.78 ± 1.60	11.46 ± 0.98	0.601	11.71 ± 1.76	11.52 ± 1.13	0.596	12.16 ± 1.65	11.80 ± 1.65	0.473
Day 2 postoperation	10.63 ± 1.02	10.86 ± 0.89	0.441	10.60 ± 1.44	10.33 ± 1.27	0.643	10.97 ± 1.45	10.90 ± 1.47	0.771
Discharge	12.35 ± 1.12	12.23 ± 1.26	0.441	12.53 ± 1.10	12.70 ± 0.55	0.145	12.85 ± 1.20	13.21 ± 0.67	0.074
Hematocrit, %									
Day 1 postoperation	35.37 + 3.15	34.81 + 4.72	0.211	30.07 ± 3.56	29.57 ± 3.34	0.279	26.33 ± 4.86	23.67 ± 3.23	0.069
Day 2 postoperation	33.23 + 2.37	31.828 + 3.45	0.175	27.72 ± 4.17	27.54 ± 3.73	0.614	26.32 ± 3.89	24.96 ± 4.59	0.409
Discharge	38.62 ± 3.52	38.31 ± 2.00	0.713	39.00 ± 0.42	38.26 ± 4.50	0.531	38.27 ± 1.71	38.77 ± 3.37	0.351

*Significance at p-value < 0.05

In group A, no significant difference was observed in the volume of allogeneic RBC transfused during and after procedure between the patients who received IAT and those who did not (P ≥ 0.05). In groups B and C, the volume of transfused allogeneic RBC was significantly reduced in patients who received IAT than those who did not, during the operation (Group B: 3.55 ± 1.27 units vs. 4.28 ± 0.71 units, P = 0.003; Group C: 5.00 ± 2.04 units vs. 8.50 ± 1.70 units, P < 0.001) and on the first day after the operation (Group B: 1.37 ± 0.68 units vs. 2.01 ± 0.76 units, P = 0.022; Group C: 2.14 ± 1.05 units vs. 2.98 ± 0.99 units, P = 0.017; Table 3).

### The RBC transfusion cost of all participants

In group A, no significant difference was observed in the allogeneic RBC transfusion-related cost between patients who received IAT and those who did not (295.39 ± 62.79 USD vs. 312.66 ± 74.15 USD, P = 0.177). In groups B and C, the allogeneic RBC transfusion-related cost in patients who received IAT was lower than that in those who did not (Group B: 375.44 ± 80.11 USD vs.468.03 ± 49.71 USD, P = 0.001; Group C: 523.36 ± 121.57 USD vs. 810.50 ± 89.01 USD, P < 0.001; Table 4).

**Table 4:** The RBC transfusion cost of all participants

	Group A			Group B			Group C		
	IAT group	No-IAT group	*P*	IAT group	No-IAT group	*P*	IAT group	No-IAT group	*P*
N	59	89		88	74		52	40	
Allogeneic RBC transfusion-related cost, USD	295.39 ± 62.79	312.66 ± 74.15	0.177	375.44 ± 80.11	468.03 ± 49.71	0.001*	523.36 ± 121.57	810.50 ± 89.01	< 0.001*
Total RBC transfusion cost, USD	539.22 ± 62.79	312.66 ± 74.15	< 0.001*	619.27 ± 80.11	468.03 ± 49.71	0.007*	767.19 ± 121.57	810.50 ± 89.01	0.015*

*Significance at p-value < 0.05

In groups A and B, patients who received IAT had significantly higher total RBC transfusion costs than those who did not (Group A: 539.22 ± 62.79 USD vs. 312.66 ± 74.15 USD, P < 0.001; Group B: 619.27 ± 80.11 USD vs. 468.03 ± 49.71 USD, P = 0.007; Table 4). In group C, significant lower total RBC transfusion cost was observed in patients who received IAT compared with that in those who did not (767.19 ± 121.57 USD vs. 810.50 ± 89.01 USD, P = 0.015; Table 4).

### Risk factors associated with massive intraoperative blood loss

A significant difference was observed between the height, weight, Cobb angle, the number of fused vertebral levels, and osteotomy pattern between the patients with intraoperative blood loss ≥ 500 to < 1,000 mL and ≥ 1,000 mL (P < 0.05). Furthermore, a significant difference was observed between the age, height, weight, Cobb angle, the number of fused vertebral levels, and osteotomy pattern between the patients with intraoperative blood loss ≥ 500 to < 1,500 mL and ≥ 1,500 mL (P < 0.05; Table 5).

**Table 5:** Risk factors associated with massive intraoperative blood loss

Volume of intraoperative blood loss	≥ 500 to < 1000 mL	≥ 1,000 mL	*P*	≥ 500 to < 1500 mL	≥ 1,500 mL	*P*
N	148	254		310	92	
Sex			0.073			0.052
Male	49	89		99	39	
Female	99	165		211	53	
Age, year	13.16 ± 2.21	12.84 ± 2.19	0.081	12.70 ± 2.08	13.44 ± 1.91	0.036*
Height, cm	146.55 ± 12.11	154.62 ± 11.09	0.020*	149.80 ± 12.09	157.55 ± 11.78	0.000*
Weight, kg	38.20 ± 8.91	43.48 ± 7.88	0.001*	39.83 ± 9.76	45.44 ± 8.12	0.001*
Cobb angle, degree	55.79 ± 0.99	63.40 ± 1.14	0.000*	58.19 ± 1.26	66.21 ± 1.32	0.000*
Number of vertebral levels fused	7.18 ± 1.10	10.46 ± 0.89	0.000*	7.98 ± 1.29	12.38 ± 0.78	0.000*
Osteotomy pattern			0.000*			0.000*
Fusion without osteotomy, n	140 (94.59)	189 (74.41)		269 (86.77)	61(66.30)	
Smith-Peterson osteotomy, n	8 (5.41)	40 (15.75)		31(10.00)	16 (17.40)	
Ponte osteotomy, n	0 (0.00)	25 (9.84)		10 (3.23)	15 (16.30)	

*Significance at p-value < 0.05

The results of the multivariate analysis revealed that the number of fused vertebral levels (volume of intraoperative blood loss ≥ 1,000 mL: odds ratio [OR] = 1.69, 95% confidence interval [CI] 1.31–3.11, P = 0.01; volume of intraoperative blood loss ≥ 1,500 mL: OR = 2.00, 95% CI 1.33–2.99, P < 0.001), and Ponte osteotomy (volume of intraoperative blood loss ≥ 1,000 mL: OR = 20.53, 95% CI 8.44–49.93, P < 0.001; volume of intraoperative blood loss ≥ 1,500 mL: OR = 23.33, 95% CI 4.40-35.37, P < 0.001) were independent risk factors for massive intraoperative blood loss (Table 6).

**Table 6:** Multivariate analysis of massive intraoperative blood loss

							95% CI for OR	
Massive intraoperative blood loss	Variables in the equation	B	SE	Wald	P	OR	Lower	Upper
≥ 1,000 mL	Height, cm	0.06	0.04	1.66	0.256	1.21	0.93	1.39
	Weight, kg	-0.02	0.03	0.20	0.357	0.97	0.91	1.24
	Cobb angle, degree	0.89	0.54	3.44	0.111	1.19	0.99	1.58
	Number of vertebral levels fused	0.89	0.22	10.51	0.010	1.69	1.31	3.11
	Osteotomy pattern							
	Fusion without osteotomy			44.49	0.000			
	Smith-Peterson osteotomy	1.19	0.54	4.71	0.063	2.29	0.72	9.66
	Ponte osteotomy	3.02	0.45	44.41	0.000	20.53	8.44	49.93
≥ 1,500 mL	Age, year	0.14	0.10	1.78	0.199	1.18	0.95	1.64
	Height, cm	0.04	0.02	2.83	0.094	1.03	0.99	1.16
	Weight, kg	-0.01	0.03	0.14	0.741	0.99	0.92	1.14
	Cobb angle, degree	0.87	0.89	4.01	0.401	1.17	0.90	1.37
	Number of vertebral levels fused	0.61	0.19	11.51	0.000	2.00	1.33	2.99
	Osteotomy pattern							
	Fusion without osteotomy			14.83	0.001			
	Smith-Peterson osteotomy	1.19	0.95	1.57	0.210	3.29	0.51	21.24
	Ponte osteotomy	3.02	0.78	14.80	0.000	23.33	4.40	35.37

**Fig. 1:**
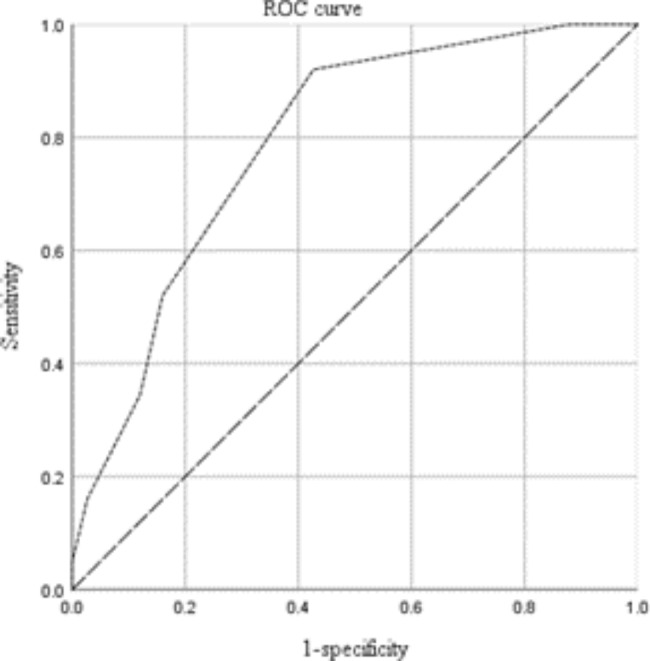
Receiver Operating Characteristic curve for predictive factors of high intraoperative blood loss (1,000 ml or greater)

**Fig. 2:**
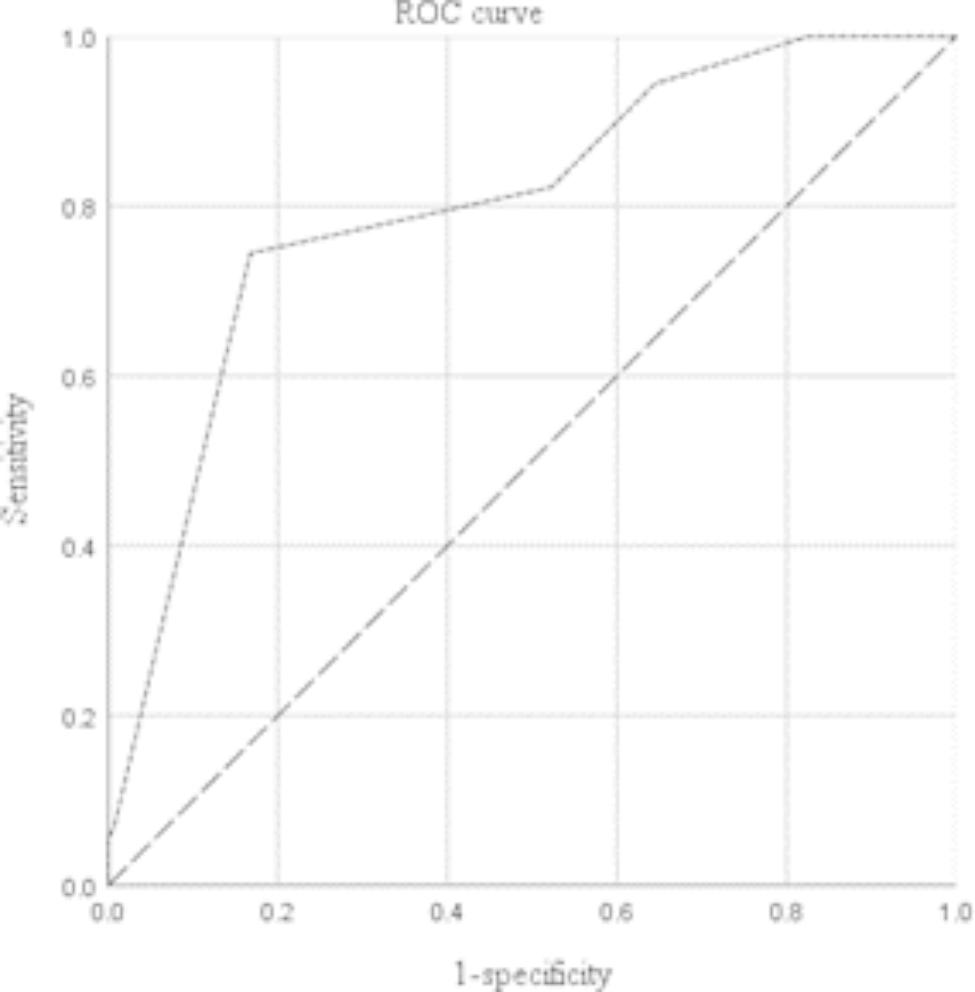
Receiver Operating Characteristic curve for predictive factors of high intraoperative blood loss (1,500 ml or greater)

ROC analysis showed that more than eight fused vertebral levels (sensitivity: 0.82, specificity: 0.58, area under the curve [AUC]: 0.79, p < 0.01) predicted 1,000 mL or greater intraoperative blood loss (Fig. 1). Additionally, more than 10 fused vertebral levels (sensitivity: 0.80, specificity: 0.52, AUC: 0.76, p < 0.01) predicted 1,500 mL or greater intraoperative blood loss (Fig. 2).

## Discussion

We discovered that the cost-effectiveness of IAT in AIS surgery is related to the amount of bleeding. According to Weiss et al., a blood loss volume > 500 mL during surgery was the threshold at which IAT cost was justified [16]. Therefore, patients with blood loss volumes less than 500 mL were excluded from our study.

The results showed that when the volume of blood loss was 500–1000 mL, IAT did not reduce the patient’s requirement for allogeneic RBC transfusion. Weiss et al. reported similar results for using IAT in scoliosis surgery. Weiss et al.’s study revealed that using IAT did not reduce the patient’s need for allogeneic RBC transfusion when the intraoperative blood loss volume averaged 733 mL [16].

In this study, IAT significantly reduced the requirement for intraoperative allogeneic RBC transfusion when the volume of blood loss was ≥ 1000 mL. Our findings are similar to those of Ersen et al. [3] and Miao et al. [15]. In Ersen et al.’s study, the average intraoperative blood loss volume was approximately 1000 mL. Their study also showed a reduced volume of allogeneic RBC transfused in patients who received IAT compared to that in the control group. In Miao et al.’s study, the intraoperative blood loss volume was approximately 2161 mL; a reduced volume of allogeneic RBC transfused in patients who used IAT was also observed. In addition, patients’ average blood volume, calculated based on weight (average 41.03 kg), was approximately 3200 mL in our study. Therefore, a blood loss volume ≥ 1000 mL was equivalent to ≥ 31% of the total blood volume. Bowen RE et al. [17] discovered that using IAT decreased the requirement for allogeneic RBC transfusion when the estimated blood loss was ≥ 30% of the total blood volume, which is consistent with our results. In addition, we found that when the volume of blood loss was ≥ 1000 mL, the use of IAT reduced the postoperative need for allogeneic RBC transfusion in scoliosis surgery. This finding was consistent with the results of Liu et al.‘s study [18], which revealed that cell salvage significantly reduced the volumes of perioperative and postoperative allogenic RBC transfusion in scoliosis surgery.

Regarding cost-effectiveness analysis, the total RBC transfusion cost in the IAT group was significantly higher than that in the no-IAT group when the blood loss volume was 500–1000 mL. This was because IAT did not decrease the patients’ requirement for allogeneic RBC transfusion. Therefore, IAT is not beneficial in AIS surgery when the blood loss volume is less than 1000 mL. However, when the blood loss volume was 1000–1500 mL, the allogeneic RBC transfusion-related cost in the IAT group was lower than that of the no-IAT group, although the total RBC transfusion cost in the IAT group was higher than that of the no-IAT group. In our study, cost of a unit of allogeneic RBC transfusion was 63.42 USD, and for using IAT per surgery it was 243.83 USD. Accordingly, when the blood loss volume was 1000–1500 mL, IAT could save approximately 93 USD off the allogeneic RBC transfusion-related cost (IAT vs. no-IAT: 375.44 ± 80.11 USD vs. 468.03 ± 49.71 USD), which was much lower than the cost of using IAT. Therefore, from an economic standpoint, we concluded that using IAT is not cost-effective when the blood loss volume is l000–1500 mL in AIS surgery. Further, when the blood loss volume was ≥ 1500 mL, significant lower allogeneic RBC transfusion-related cost and total RBC transfusion cost were observed in patients who received IAT compared to that in those who did not. Therefore, when the blood loss volume was ≥ 1500 mL, IAT was cost-effective, drastically reducing the demand for allogeneic RBC and total RBC transfusion cost.

The results of the multivariate analysis revealed that the number of fused vertebral levels was an independent risk factor for massive intraoperative blood loss, which is consistent with Li et al. [19] and Yu et al. [20]. Li et al. discovered that in patients with AIS undergoing posterior internal fixation surgery, the operative time and number of surgical fixation segments were risk factors for total blood loss. Yu et al. discovered that fusing more than six levels was a risk factor for massive intraoperative blood loss. An increase in the number of fused vertebral levels increases the length of the surgical incision and amount of muscle dissection [21]. The ROC analysis revealed that the number of fused vertebral levels of more than eight predicted 1,000 mL or greater intraoperative blood loss, while the number of fused vertebral levels of more than 10 predicted 1,500 mL or greater intraoperative blood loss. This suggests that we can predict the amount of bleeding based on the number of fused vertebral levels and further judge whether to use IAT. In AIS surgery, when the number of fused vertebrae exceeds eight, the intraoperative blood loss is greater than 1,000 mL; in this case, using IAT would reduce the need for infusion of allogeneic RBCs. Furthermore, when more than 10 vertebral levels were fused, the intraoperative blood loss was greater than 1,500 mL; in this case, IAT was cost-effective, drastically reducing the demand for allogeneic RBCs and total RBC transfusion cost.

In addition, we found that Ponte osteotomy was also an independent risk factor for massive intraoperative blood loss, which was consistent with the results of most previous studies. Koerner et al. [22] found that in patients with AIS undergoing posterior spinal fusion with instrumentation, Ponte osteotomy increased all measures of intraoperative blood loss and need for transfusion. Wang et al. [23] discovered that Ponte osteotomy could obtain better coronal correction and sagittal contour restoration in patients with AIS and hypokyphosis; however, Ponte osteotomies might lead to more intraoperative blood loss and longer operation time.

Currently, the results of studies on the effect of Cobb angle on intraoperative blood loss are inconsistent. Yu et al. [20] and Song et al. [24] found that large preoperative Cobb angle was related to massive intraoperative blood loss. Large preoperative Cobb angle and correction angle require different degrees of orthopaedic osteotomy and more internal fixation segments, which increases the operative difficulty and time [20]. In this study, although Cobb angle was a potential risk factor of massive intraoperative blood loss, multiple linear regression analysis showed that the Cobb angle was not a risk factor. Our finding was consistent with those of Li et al. [19] and Tang et al. [25]. Future studies with larger sample sizes are needed to resolve this disagreement.

This study had two limitations. First, allogeneic RBC products and IAT costs were based on the Chinese charging standard for medical treatment (RBC/IAT: 63.42 USD/243.83 USD). In the United States and Europe, the direct cost of a unit of allogeneic blood varies from 386 to 717 USD, and the cost of performing IAT varies from 240 to 512 USD per case [10, 26–28]. The costs of allogeneic RBC products in the United States and Europe are higher than that reported in our study (China). Thus, if the cost of using IAT were reduced to less than 243.83 USD, whether in China, the United States, or Europe, IAT in AIS surgery would be cost-effective when the blood loss volume was over 1500 mL. Second, our study was a single-centre retrospective study, which may not completely exclude other potential clinical factors, such as administration of erythropoiesis stimulating agents (ESAs) and iron during the perioperative period, that might affect the overall outcome. Researchers have shown that combined administration of erythropoiesis stimulating agents (ESAs) and iron reduces the number of red blood cell transfusions in the postoperative period [29, 30].

## Conclusions

In scoliosis surgery, the cost-effectiveness of IAT is related to the volume of blood loss. In this respect, in our study when the blood loss volume was over 1500 mL, IAT was cost-effective, drastically reducing the demand for allogeneic RBC and total RBC transfusion cost. The number of fused vertebral levels and Ponte osteotomy were independent risk factors for massive intraoperative blood loss. In addition, the number of fused vertebral levels could predict massive intraoperative blood loss; when the number of fused vertebrae exceed eight, the intraoperative blood loss would be greater than 1,000 mL, and when more than 10 vertebral levels are fused, the intraoperative blood loss would be greater than 1,500 mL. This study resolves the controversy regarding the cost-effectiveness of IAT and provides suggestions for its rational use in clinical practice.

## Data Availability

The datasets used and analysed during the current study are available from the corresponding author on reasonable request. Competing Interests. The authors have no competing interests to declare.

## Abbreviations

IAT: Intraoperative autologous transfusion
AIS: Adolescent idiopathic scoliosis
RBC: Red blood cells
ROC: Receiver operating characteristic
SD: Standard deviations
